# Supplementary figures and images for: FM3VCF: A software library for accelerating the loading of large VCF files in genotype data analyses
Source: PLoS One. 2025 Jun 4;20(6):e0324430. doi: 10.1371/journal.pone.0324430 (PMC12136311; doi:10.1371/journal.pone.0324430)

**a**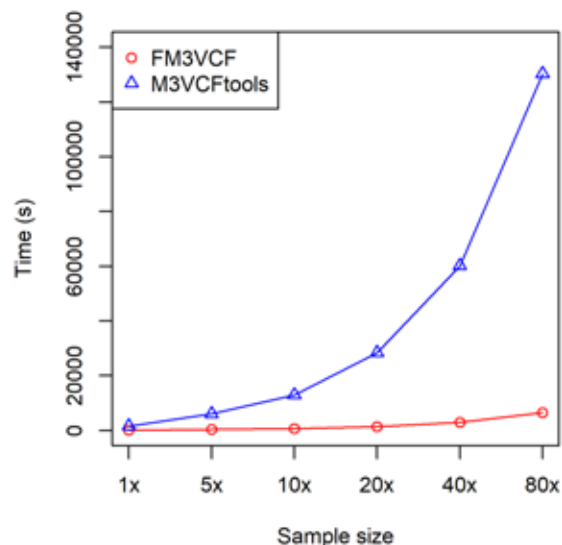**b**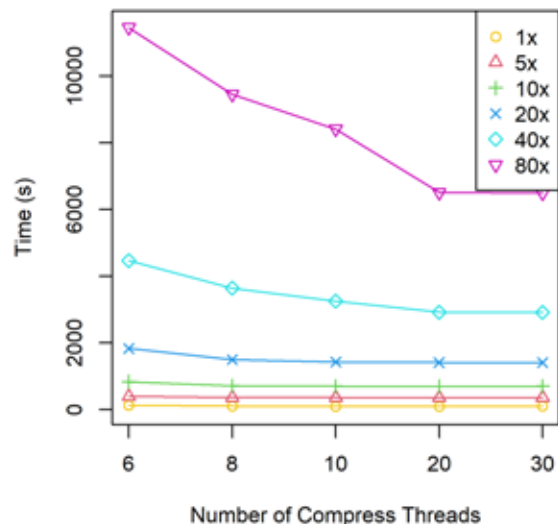**c**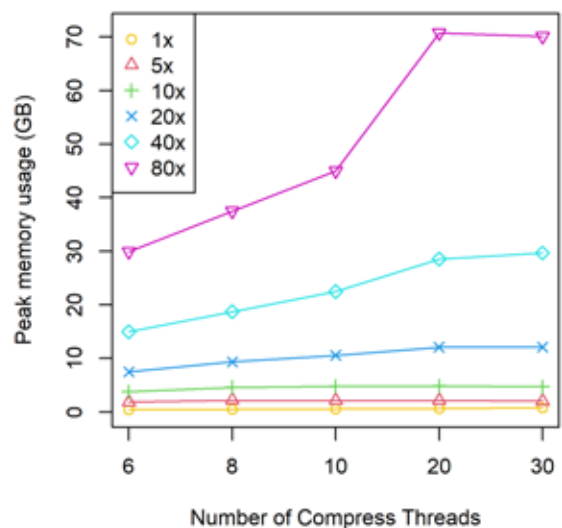**d**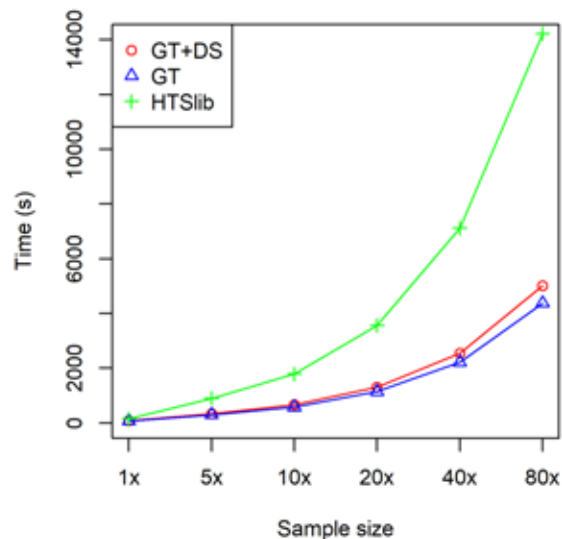

Supplement: S1 Fig — The dataset is derived from chromosome 22 of the 1000 Genomes Project, containing 1,092 samples and 494,328 variants (http://ftp.1000genomes.ebi.ac.uk/vol1/ftp/release/20110521/ALL.chr22.phase1_release_v3.20101123.snps_indels_svs.genotypes.vcf.gz). (a) The figure shows the compression time for VCF files of different sizes. The blue line represents the results obtained using m3VCFtools, while the red line represents the results obtained using FM3VCF. (b) Multi-thread computation time for FM3VCF. The results for sample sizes of 80x, 40x, 20x, 10x, 5x, and 1x are represented by the purple, cyan, blue, green, red, and orange lines, respectively. (c) Multi-thread peak memory usage by FM3VCF. The results for sample sizes of 80x, 40x, 20x, 10x, 5x, and 1x are represented by the purple, cyan, blue, green, red, and orange lines, respectively. (d) Reading and parsing times for VCF files of different sizes. The green line corresponds to the reading time for different sample sizes using HTSlib. The red line represents the time required for parsing GT and DS by FM3VCF, while the blue line shows the time needed for parsing GT by FM3VCF. (PDF) [file pone.0324430.s001.pdf]
